# Reduced production and uptake of lactate are essential for the ability of WNT5A signaling to inhibit breast cancer cell migration and invasion

**DOI:** 10.18632/oncotarget.17277

**Published:** 2017-04-20

**Authors:** Chandra Prakash Prasad, Katja Södergren, Tommy Andersson

**Affiliations:** ^1^ Cell and Experimental Pathology, Department of Translational Medicine, Lund University, Clinical Research Centre, Skåne University Hospital, SE-20502 Malmö, Sweden

**Keywords:** WNT5A, breast cancer cells, PFKP, lactate, cell migration and invasion

## Abstract

Here we investigated the impact of WNT5A signaling on aerobic glycolysis and evaluated its effects on breast cancer cell migration/invasion. WNT5A signaling reduced migration and lactate production and caused selective down-regulation of the glycolytic enzyme phosphofructokinase platelet-type (PFKP). These events occurred in parallel with a WNT5A-induced inhibition of β-catenin signaling. Support for essential involvement of β-catenin and PFKP in lactate production and migration/invasion was obtained by siRNA knockdown of their expression. To also explore the effect of non-tumor cell-derived lactate, we added exogenous lactate to the cells and noted an increase in migration that was significantly impaired by recombinant WNT5A in parallel with a down-regulation of the lactate transporter monocarboxylate transporter 1 (MCT1). Interestingly enough, the drug-candidate Foxy5 (WNT5A-mimic hexapeptide) also inhibited breast cancer cell migration in the presence of exogenous lactate, suggesting a therapeutic potential for Foxy5 in managing breast tumors with high glycolytic activity. Overall, we demonstrated that WNT5A signaling (via a β-catenin-PFKP axis) reduces lactate production and lowers the expression of MCT1, a carrier mediating the uptake of lactate from the tumor microenvironment. These effects of WNT5A are essential for its ability to impair breast cancer migration/invasion even in an environment with elevated lactate levels.

## INTRODUCTION

Tumor growth depends on glucose uptake from the extracellular environment and its glycolytic breakdown resulting in accumulation of lactate even if O_2_ is present. This metabolic phenomenon is known as the ‘Warburg effect’ [1]. Although aerobic glycolysis is a more inefficient process compared to oxidative phosphorylation, cancer cells compensate for this by increasing their rate of glucose flux. The increased aerobic glycolysis results in accumulation of lactate both inside and outside of the tumor cells, i.e., in the extracellular tumor microenvironment. In addition, stromal cells in the tumor microenvironment can also contribute to the accumulation of lactate and thereby exposing tumor cells to a high lactate concentrations [2–5]. Clinically, the lactate concentration in tumors has been estimated to be as high as 40 mM compared to the physiologic normal range of 0.5–2 mM [6].

Both β-catenin-dependent and β-catenin-independent WNT signaling have been implicated as mediators of cancer cell metabolism [7]. In breast cancer cells, β-catenin-dependent WNT signaling induces aerobic glycolysis in breast cancer via down-regulation of cytochrome *c* oxidase, thereby suppressing mitochondrial respiration [8]. c-Myc, a transcriptional target of WNT β-catenin signaling, is known to upregulate key rate-limiting glycolytic genes, e.g., *GLUT-1*, *LDH* and *PKM2*, thereby stimulating aerobic glycolysis in cancer cells [9, 10]. These findings are in accordance with the demonstrated role of β-catenin-dependent signaling in the development and progression of breast cancer [11, 12]. In addition, there is direct and indirect evidence that suggests an essential role of β-catenin-independent WNT signaling in the regulation of cancer cell metabolism [7]. The WNT5A ligand may accomplish its effect on cancer cell metabolism directly or by antagonizing β-catenin-dependent signaling [7]. The latter mechanism has been shown to be a part of its role as a tumor suppressor in colon cancer, ovarian cancer and breast cancer [13–15].

WNT5A is a secreted glycoprotein that binds to specific receptor/co-receptor complexes to initiate intracellular signaling cascades that results in regulation of different cellular processes, including cell proliferation, differentiation and migration [16–18]. Distinct expression of WNT5A has been reported in various cancers. In breast cancer, loss of WNT5A expression has been associated with early relapse and unfavorable prognosis [19–21]. A similar tumor suppressive role of WNT5A has also been suggested in colon cancer and prostate cancer [22–25]. In contrast, WNT5A has a tumor-promoting role in melanoma where it has been shown to escalate cell migration and invasion, thereby promoting melanoma metastasis [26–28]. Recently, Sherwood *et al*. demonstrated that WNT5A can reprogram tumor cells and that these effects are diverse and context dependent [29]. For example, in contrast to melanoma cells, WNT5A-treated breast cancer cells do not respond with an increase in aerobic glycolysis but instead with a significant increase in their oxygen consumption rate (OCR) [29].

Consequently, based on the previous report by Sherwood *et al*., we expanded the metabolic investigation of the consequences of WNT5A signaling in two metastatic breast cancer cell lines (MDA-MB-468 and MDA-MB-231) in the present study. We first transfected both breast cancer cell lines with a WNT5A plasmid to study the long-term effects of WNT5A signaling in an environment that mimics *in vivo* conditions. We also stimulated the breast cancer cells lines with recombinant WNT5A (rWNT5A) and Foxy5, a WNT5A-mimic peptide that is presently in a clinical phase 1b study. Our present findings revealed the mechanisms whereby WNT5A signaling reduces lactate production and the uptake of lactate from the extracellular microenvironment, and they also demonstrated that the WNT5A-induced metabolic changes are essential for its ability to impair breast cancer cell migration and invasion. Due to the inhibition of breast cancer cell migration, even in the presence of extracellular lactate, the WNT5A-mimic peptide, Foxy5, is implicated as a potential therapeutic agent in the treatment of extremely glycolytic and aggressive breast cancers.

## RESULTS

### WNT5A signaling inhibits lactate production and cell migration without affecting cell proliferation

**Figure 1:**
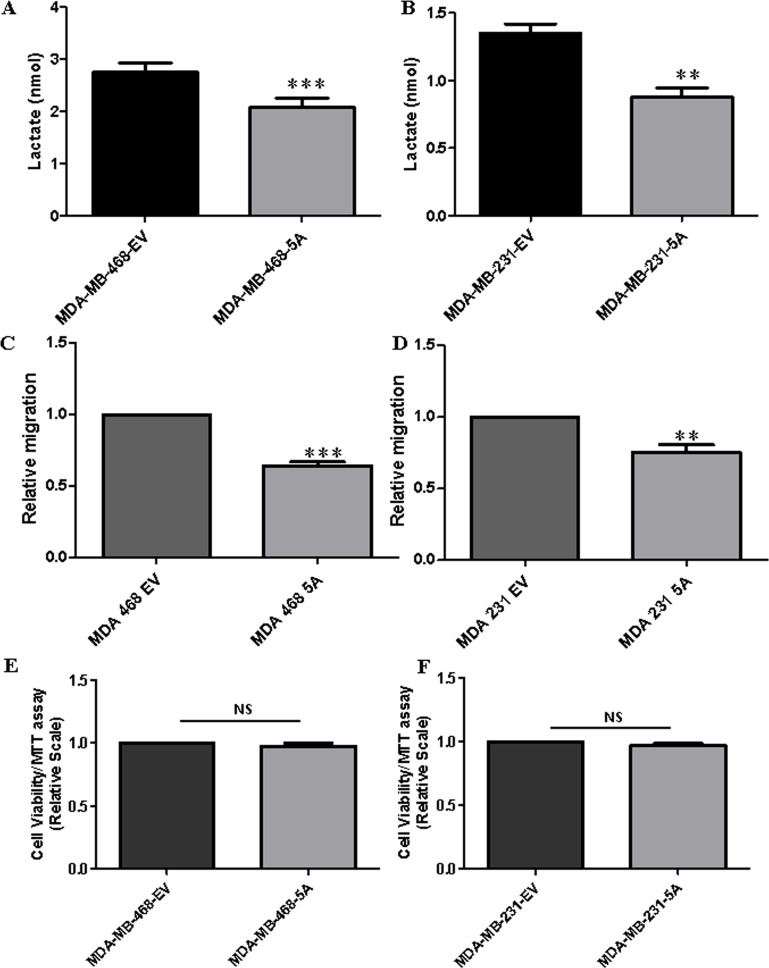
WNT5A signaling inhibits lactate production and cell migration but not cell proliferation in breast cancer cells Cell culture media lactate measurements for the (**A**) MDA-MB-468-5A and (**B**) MDA-MB-231-5A WNT5A-expressing breast cancer cells compared to empty vector-expressing cells at 72 h. All experiments with WNT5A transfected breast cancer cells were performed for 72 h (end point) to allow sufficient time for secretion of WNT5A. All error bars represent the standard error of the mean (*n* = 4). **p = 0.01, ***p = 0.001. Transwell migration assays were performed using (**C**) MDA-MB-468-5A and (**D**) MDA-MB-231-5A WNT5A-expressing breast cancer cells compared to empty vector-expressing cells at 72 h. All error bars represent the standard error of the mean (*n* = 4). ***p = 0.01, ***p = 0.001. A MTT cell viability assay was performed in the (**E**) MDA-MB-468-5A and (**F**) MDA-MB-231-5A WNT5A-expressing breast cancer cells for 72 h as described in the Materials and Methods section. The results were evaluated at 570 nm using a multi-well plate reader. All error bars represent the standard error of the mean (*n* = 4). NS=Non-Significant.

Previously, Sherwood *et al*. demonstrated that WNT5A signaling positively regulates aerobic glycolysis in melanomas, thereby making melanoma cells more migratory and invasive [29]. In the same study, the authors’ also demonstrated that rWNTA-treated MDA-MB-468 breast cancer cells show induced oxidative phosphorylation but no changes in lactate secretion [29]. In the present study, we performed a more thorough investigation of the role of WNT5A signaling on aerobic glycolysis in breast cancer cells using several different approaches. Although rWNT5A clearly triggers WNT5A signaling in breast cancer cells, the stability of rWNT5A in cell culture conditions remains unknown. A potential variability in the concentration of rWNT5A might affect its regulation of aerobic glycolysis and oxidative phosphorylation. Thus, to address this problem, we transfected two metastatic breast cancer cell lines (MDA-MB-468 and MDA-MB-231) with a WNT5A plasmid as these cells have no endogenous WNT5A expression (Supplementary Figure 1). Both breast cancer cell lines expressing the WNT5A plasmid (MDA-MB-468-5A and MDA-MB-231-5A) showed significant decreases in lactate production after 72 h compared to the respective empty vector (EV)-transfected control cells (Figure 1A and 1B). These findings clearly demonstrated that WNT5A signaling inhibits lactate production in breast cancer cells, which is in direct contrast to its effect in melanoma cells. Using the same experimental conditions, we investigated parallel effects on migration and proliferation of the breast cancer cells via Transwell migration and MTT assays. Compared to their respective controls (EV), migration was significantly decreased in WNT5A-expressing breast cancer cells (Figure 1C and 1D). Morphologically, WNT5A expressing breast cancer cells exhibited less migration relevant membrane protrusions, as compared to control EV transfected cells (Supplementary Figure 2). WNT5A signaling did not affect breast cancer cell growth (Figure 1E and 1F). These experiments suggested a potential relation between lactate secretion and cell migration in breast cancer cells.

### Phosphofructokinase platelet-type (PFKP) expression predicts overall survival in breast cancer patients

**Figure 2:**
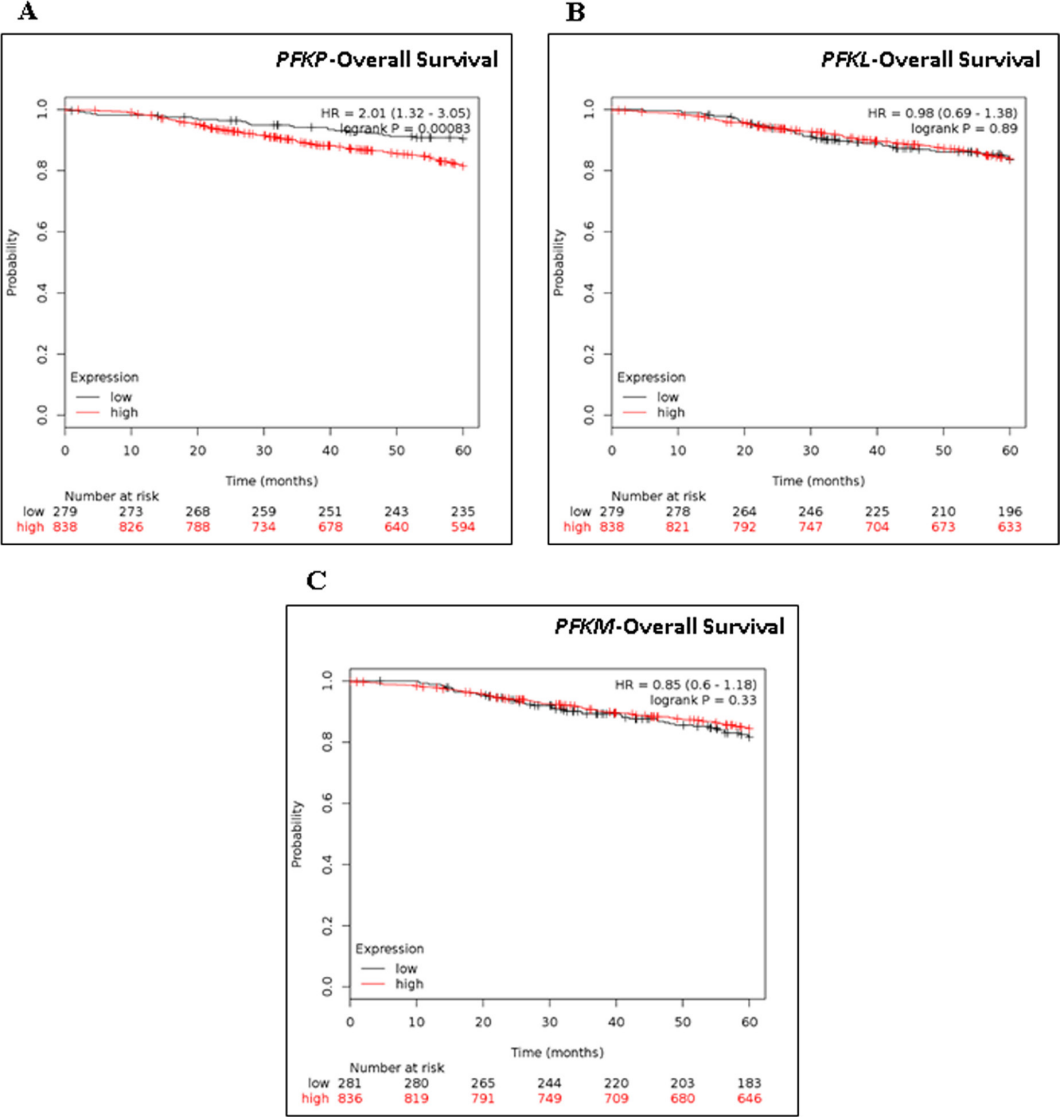
Kaplan-Meier Survival analysis of PFKP, PKFL, and PKFM in breast cancer patients Kaplan-Meir survival analysis of (**A**) *PFKP*, (**B**) *PKFL*, and (**C**) *PKFM* mRNA in 1117 breast cancer patients with Kaplan-Meier Plotter. Auto select best cutoff was chosen in the analysis. Cutoff value was 558. Expression range of the probe was 8–13211. Hazard ratio (HR) and Log-rank *p* values are shown.

Phosphofructokinase (PFK) plays a key role in regulating glycolytic flux by converting fructose 6-phosphate to fructose 1,6-bisphosphate, a committed step in the glycolytic pathway [30]. PFK is a complex tetrameric enzyme that exists in three isoforms as follows: liver (PFKL), muscle (PFKM), and platelet (PFKP). To explore the relevance of these PFK isoforms in breast cancer, we investigated how their respective expression related to breast cancer patient survival by using Kaplan-Meier survival analysis. Using online meta-analysis software, gene expression profiles of *PFKP*, *PFKL* and *PFKM* derived from GEO (Affymetrix microarrays only), EGA and TCGA data sets were generated using 1117 breast tumor samples as described by Gyorffy *et al*. [31]. Of the three PFK isoforms, the Kaplan–Meier survival analyses showed that high *PFKP* expression correlated with decreased patient survival (HR = 2.01; *p* = 0.00083), whereas the two other PFK isoforms (*PFKL* and *PFKM*) did not (Figure 2A–2C). Overall, these results indicate that the expression of PFKP not only relates to the glycolytic activity but also to breast cancer patient prognosis.

### WNT5A regulates PFKP protein expression in breast cancer cells

**Figure 3:**
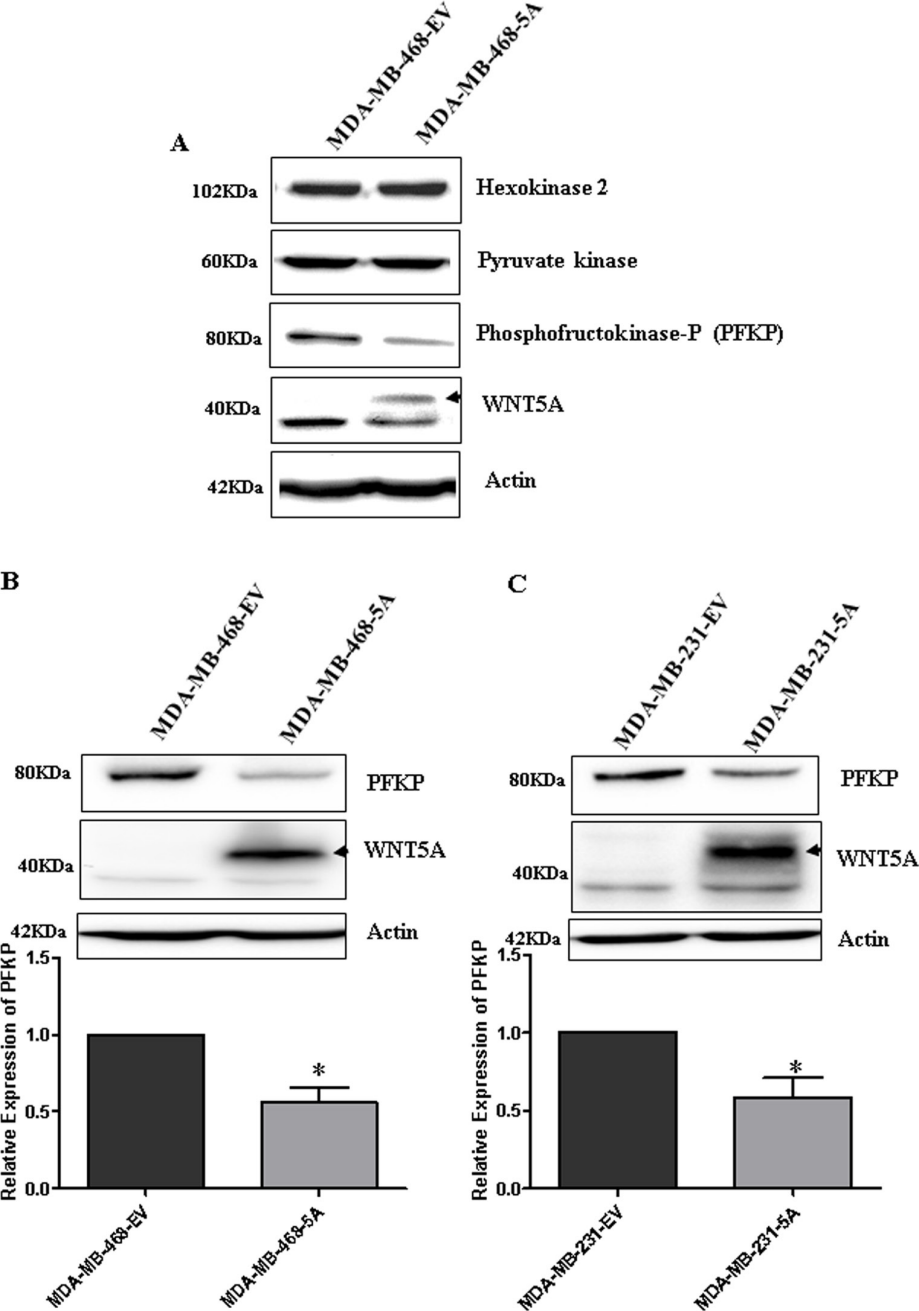
WNT5A signaling inhibits PFKP expression in breast cancer cells (**A**) Representative Western blot showing changes in key glycolytic markers in the MDA-MB-468-5A WNT5A-expressing breast cancer cells compared to empty vector-expressing cells (MDA-MB-468-EV). (**B**) Representative Western blots and quantification of PFKP protein in MDA MB-468-5A cells compared to MDA-MB-468-EV cells. PFKP levels were quantified by calculating integrated densitometric values and normalizing them to actin levels. (**C**) Representative Western blots and quantification of PFKP protein in MDA-MB-231-5A cells compared to MDA-MB-231-EV cells. PFKP levels were quantified by calculating integrated densitometric values and normalizing them to actin levels. All error bars represent the standard error of the mean (*n* = 3). *p < 0.05.

Our initial findings revealed that WNT5A signaling impairs lactate production in breast cancer cells and that *PFKP* expression relates to prognosis of breast cancer patients. These results made us investigate if these findings occurred simultaneously with a WNT5A-induced altered protein expression of not only PFKP but also of two additional key glycolytic proteins, Hexokinase II (HK) and pyruvate kinase (PK), in breast cancer cells. We have in the present study focused our interest on the potential roles of enzymes that are designated as critical regulators of glycolysis [32–35]. However, it is important to underline that this does not exclude contribution of other enzymes in the regulation of lactate production. Using Western blotting, we investigated the expression of HK, PK and PFKP in MDAMB-468-5A cells, as these glycolytic enzymes are crucially involved in the production of lactate and play essential roles in breast cancer progression [36–38]. Of these three enzymes, only the expression of PFKP was decreased in MDA-MB-468-5A cells as compared to control MDA-MB-468-EV cells (Figure 3A). WNT5A expression significantly reduced PFKP expression in both breast cancer cell lines (i.e., MDA-MB-468-5A and MDA-MB-231-5A) compared to their respective EV-transfected control cells (Figure 3B and 3C). The down-regulation of PFKP, a key glycolytic enzyme, by WNT5A signaling correlates with the ability of WNT5A to decrease lactate secretion in breast cancer cells.

### PFKP knockdown inhibits lactate production and impairs breast cancer cell migration

**Figure 4:**
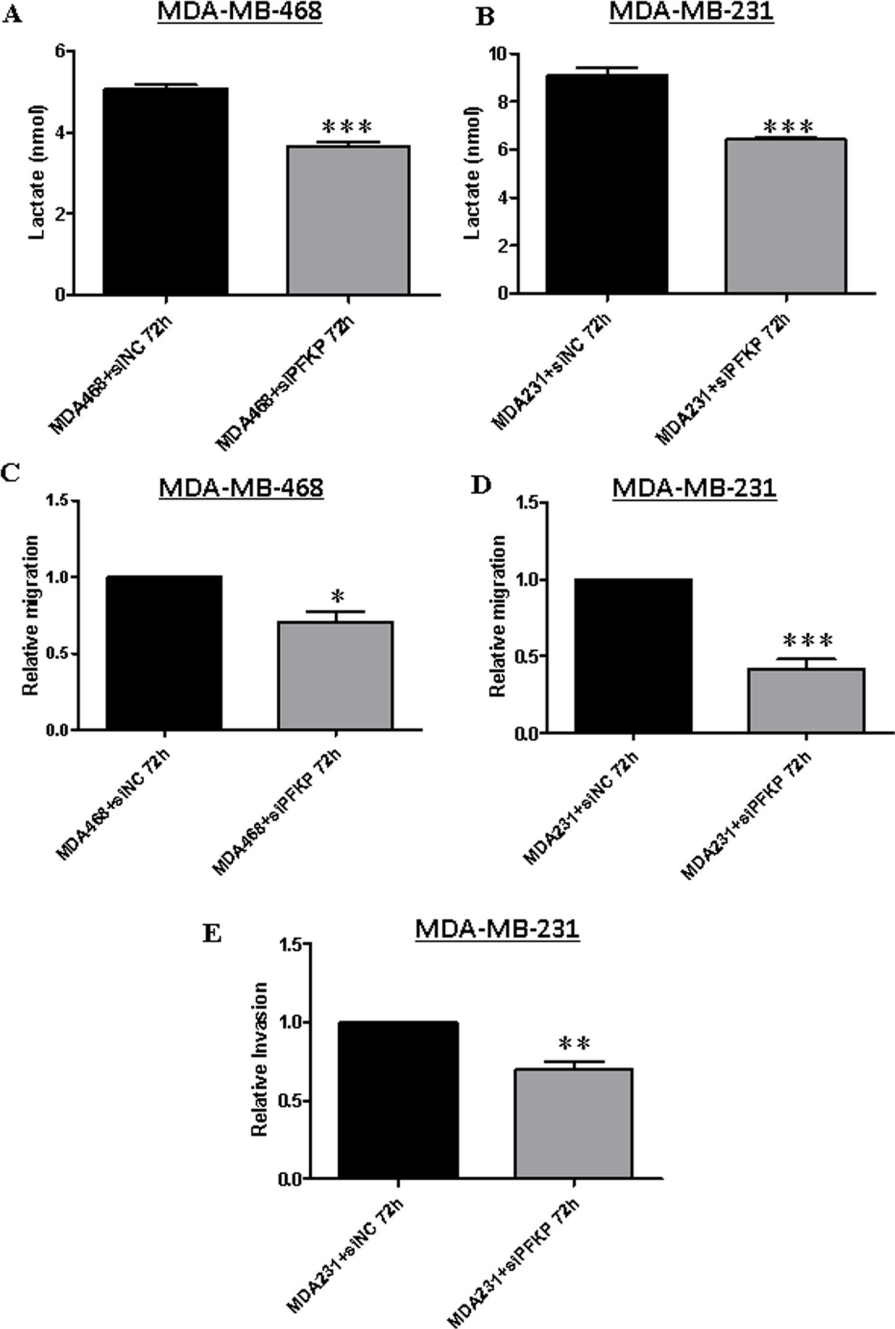
PFKP knockdown inhibits lactate production and impairs breast cancer cell migration and invasion Extracellular lactate measurements in (**A**) MDA-MB-468 and (**B**) MDA-MB-231 breast cancer cells transiently transfected with PFKP siRNA compared to control NC siRNA as described in the Materials and Methods section. All error bars represent the standard error of the mean (*n* = 4). ***p = 0.001. (**C**) MDA-MB-468 and (**D**) MDA-MB-231 cells were transiently transfected with PFKP siRNA and subjected to transwell migration analysis as described in the Materials and Methods section. All error bars represent the standard error of the mean (*n* = 5). *p < 0.05, ***p = 0.001. (**E**) MDA-MB-231 cells were transiently transfected with PFKP siRNA and subjected to transwell invasion analysis as described in the Materials and Methods section. All error bars represent the standard error of the mean (*n* = 4). **p = 0.01.

To further investigate the functional role of PFKP in breast cancer cells, we knocked down PFKP expression using specific siRNAs (as described in the Materials and Methods section). For siRNA efficiency and specificity experiments, we transiently transfected parental MDA-MB-231 cells with 25, 50 or 100 nM PFKP siRNA for 48 h and analyzed the effects on PFKP expression by Western blotting (Supplementary Figure 3). Of the three concentrations tested, we selected 25 nM for the subsequent experiments. We next examined if PFKP silencing affected lactate production in parental breast cancer cell lines. Both MDA-MB-468 and MDA-MB-231 cells were transfected with 25 nM PFKP siRNA, and lactate production was then analyzed after 72 h. Significant reductions in lactate production were observed in both PFKP-silenced breast cancer cell lines (MDA-MB-468 and MDA-MB-231) as compared to their respective control cells (NC) (Figure 4A and 4B). These results support the notion that the WNT5A-mediated decrease in lactate production in breast cancer cells is due to the WNT5A-induced reduced expression of PFKP protein. To investigate the functional role of PFKP in breast cancer cells, we next investigated how the reduced expression of PFKP affects breast cancer cell migration and invasion. PFKP-silenced MDA-MB-468 (Figure 4C) and MDA-MB-231 (Figure 4D) cells showed significantly impaired migration compared to their respective controls. For the invasion experiments, we selected the highly invasive MDA-MB-231 cells over the less invasive MDA-MB-468 cells. In accordance with the migration results, PFKP siRNA treatment of MDA-MB-231 cells resulted in significant inhibition of their invading capacity compared to cells treated with negative control (NC) siRNA (Figure 4E). These results indicated that PFKP expression positively regulates not only lactate production but also breast cancer cell migration and invasion.

### WNT5A regulates PFKP expression through β-catenin

**Figure 5:**
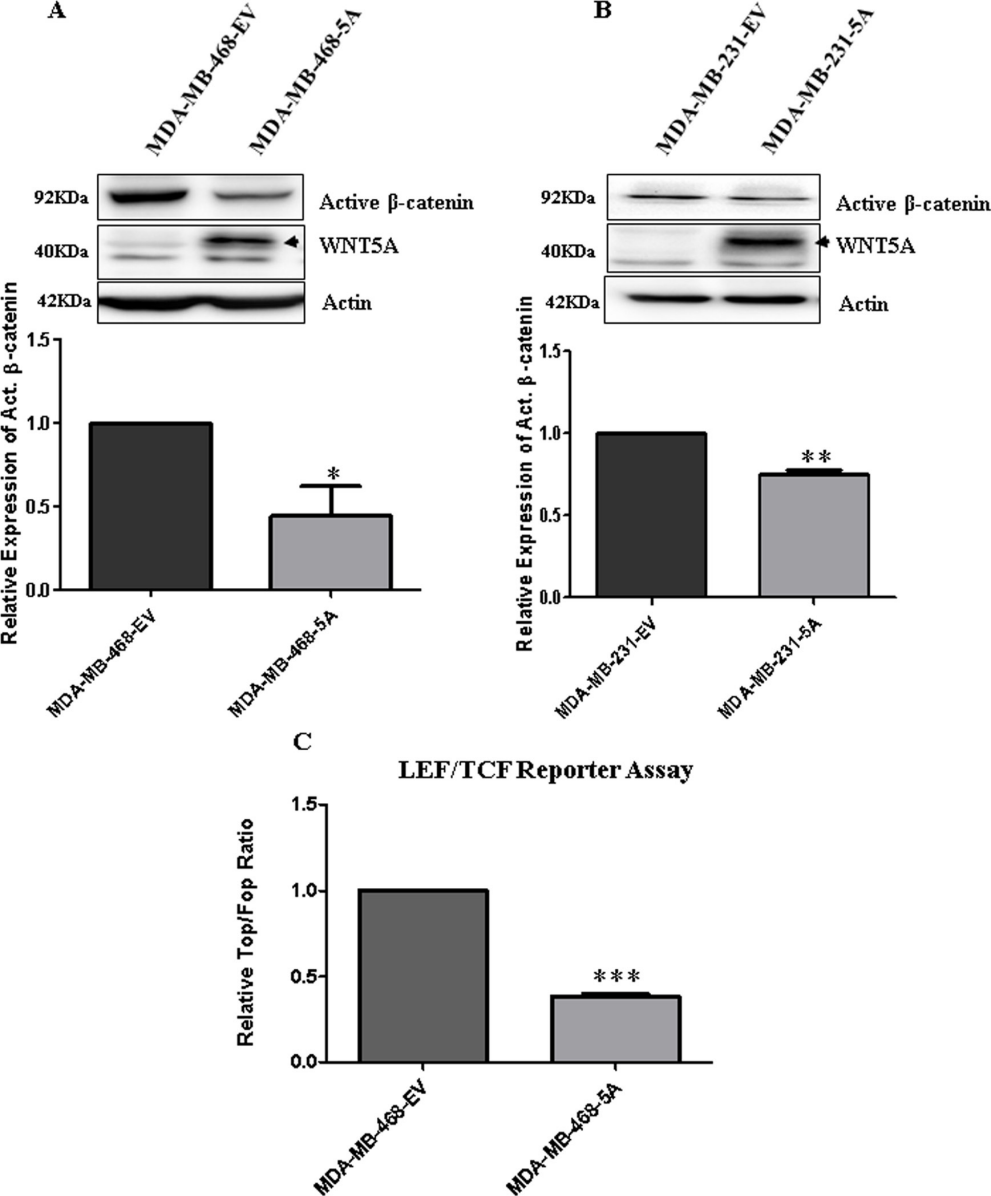
WNT5A signaling inhibits β-catenin expression in breast cancer cells (**A**) Representative Western blots and quantification of non-phospho (Active) β-catenin protein in MDA MB-468-5A cells compared to MDA-MB-468-EV cells. Active β-catenin levels were quantified by calculating integrated densitometric values and normalizing them to actin levels. All error bars represent the standard error of the mean (*n* = 3). *p < 0.05. (**B**) Representative Western blots and quantification of non-phospho (Active) β-catenin protein in MDA-MB-231-5A cells compared to MDA-MB-231-EV cells. Active β-catenin levels were quantified by calculating integrated densitometric values and normalizing them to actin levels. All error bars represent the standard error of the mean (*n* = 3). **p = 0.01. (**C**) Relative luciferase activity (TOP Flash over FOP Flash) was measured in the MDA-MB-468-5A WNT5A-expressing breast cancer cells compared to empty vector-expressing cells (MDA-MB-468-EV). All error bars represent the standard error of the mean (*n* = 4). ***p = 0.001.

Because we have shown that WNT5A inhibits PFKP expression in breast cancer cells and that PFKP directly regulates aerobic glycolysis, migration and invasion of breast cancer cells, we investigated the mechanism of WNT5A-mediated regulation of PFKP expression in breast cancer cells. We examined the role of β-catenin signaling for the following two reasons: 1) active β-catenin signaling positively regulates aerobic glycolysis in cancers, including breast tumors [8, 39]; and 2) WNT5A expression inhibits ERK1/2 signaling in breast cancer cells [40], and ERK1/2 signaling has been suggested to activate WNT β-catenin signaling [41, 42]. Hence, we analyzed the expression of active (non-phosphorylated) β-catenin in the two WNT5A-expressing breast cancer cell lines. Significant reductions in the expression of active β-catenin were observed in the cell lysates of both WNT5A-expressing breast cancer cell lines (Figure 5A and 5B) as compared to their respective control EV cells. Moreover, significant inhibition of LEF/TCF reporter activity was found in MDA-MB-468-5A cells compared to MDA-MB-468-EV cells (Figure 5C), thereby indicating that WNT5A signaling impairs β-catenin signaling in breast cancer cells. To investigate if the WNT5A-mediated inhibition of β-catenin is mediated through altered ERK1/2 signaling, we next treated the breast cancer cell lines with U0126, a highly selective inhibitor of both MEK1 and MEK2 resulting in impaired ERK signaling (Supplementary Figure 4A and 4B). Exposure to U0126 for 24 h reduced active β-catenin protein expression in parental MDA-MB468 and MDA-MB231 breast cancer cells (Supplementary Figure 4A and 4B), demonstrating that β-catenin is a downstream target of ERK1/2 suggesting that WNT5A-induced inhibition of ERK1/2 activity is a possible cause of its inhibitory effect on β-catenin in these cells. Alternatively, U0126 might alter the level of P-LRP6 in WNT5A expressing cells and thereby regulate β-catenin signaling.

We next investigated the effect of β-catenin inhibition on aerobic glycolysis by analyzing lactate production in these breast cancer cells. For the experiments, we used XAV939, a potent tankyrase (TNKS) inhibitor that antagonizes β-catenin-dependent WNT signaling via stimulation of β-catenin degradation and stabilization of axin [43]. Treatment of MDA-MB-468 and MDA-MB-231 cells for 24 h with XAV939 (10 μM) resulted in a significant decrease in lactate production as compared to vehicle (DMSO)-treated cells (Supplementary Figure 4C and 4D), demonstrating that β-catenin signaling positively regulates aerobic glycolysis in these breast cancer cells.

**Figure 6:**
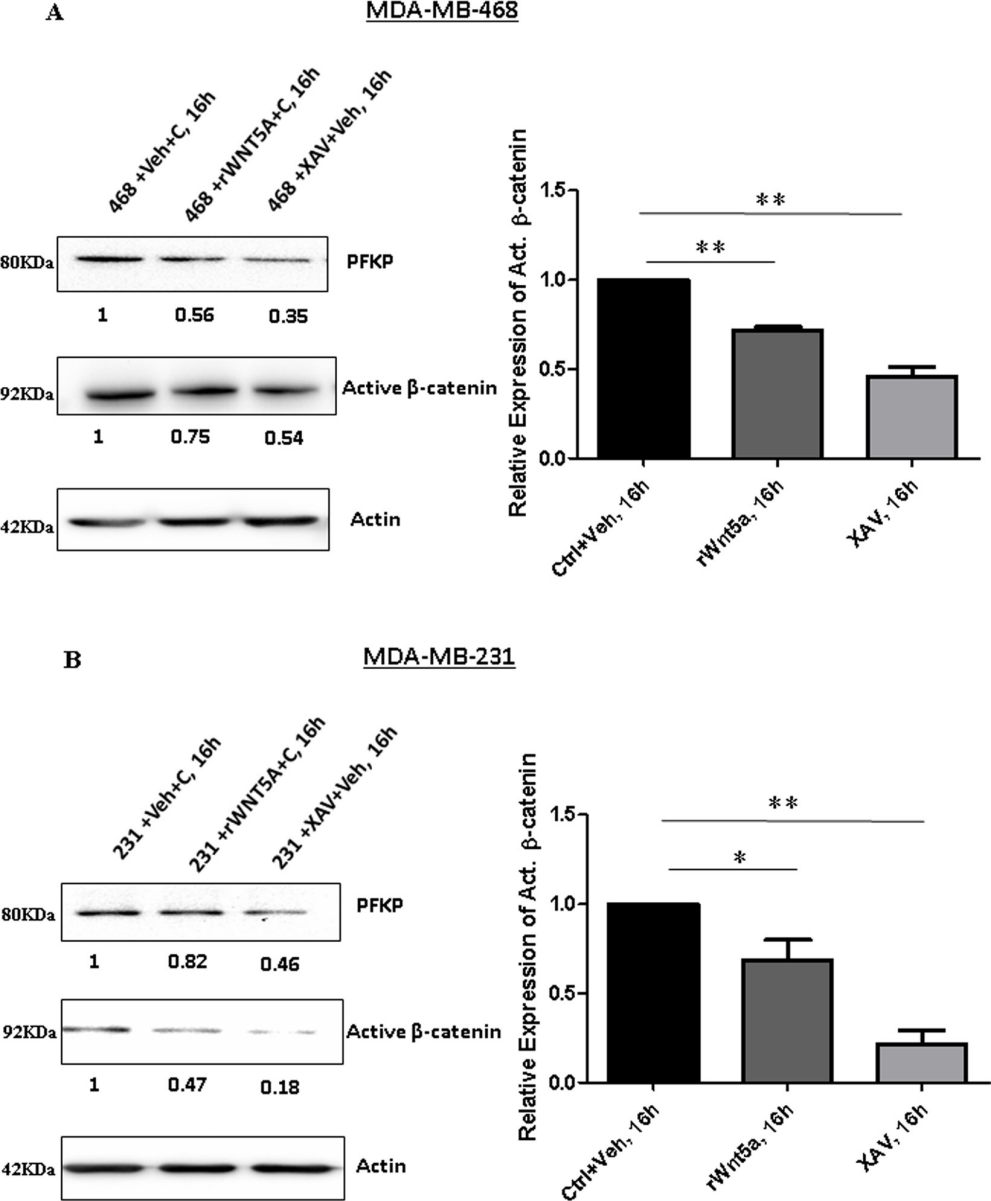
Inhibition of β-catenin results in reduced expression of PFKP in breast cancer cells Parental breast cancer cells were treated with recombinant WNT5A (rWNT5A; 0.4 μg/ml) and XAV939 (β-catenin inhibitor; 10 μM) for 16 h. (**A**) Representative Western blot (*left panel*) demonstrating the relative expression levels of PFKP and active β-catenin protein in MDA-MB-468 cells after treatment with rWNT5A and XAV. Actin served as a loading control. Active β-catenin levels for the experiments were quantified by calculating integrated densitometric values and normalizing them to actin levels (*right panel*). All error bars represent the standard error of the mean (*n* = 3). **p = 0.01. (**B**) Representative Western blot (*left panel*) demonstrating the relative expression levels of PFKP and active β-catenin protein in MDA-MB-231 cells after treatment with rWNT5A and XAV. Actin served as a loading control. Active β-catenin levels for the experiments were quantified by calculating integrated densitometric values and normalizing them to actin levels (*right panel*). All error bars represent the standard error of the mean (*n* = 3). *p < 0.05, **p = 0.01.

We next tested if direct inhibition of β-catenin signaling in breast cancer cells will result in decreased PFKP protein expression. We treated parental MDA-MB-468 and MDA-MB-231 cells with rWNT5A and XAV939 independently and analyzed PFKP expression by Western blotting. Treatment with either rWNT5A or XAV939 resulted in significant decreases in the expression of active β-catenin and PFKP in MDA-MB-468 (Figure 6A) and MDA-MB-231 (Figure 6B) cells. These data suggested that β-catenin positively regulates PFKP expression and thereby aerobic glycolysis in breast cancer cells.

### WNT5A signaling inhibits breast cancer cell migration even in the presence of extracellular lactate

**Figure 7:**
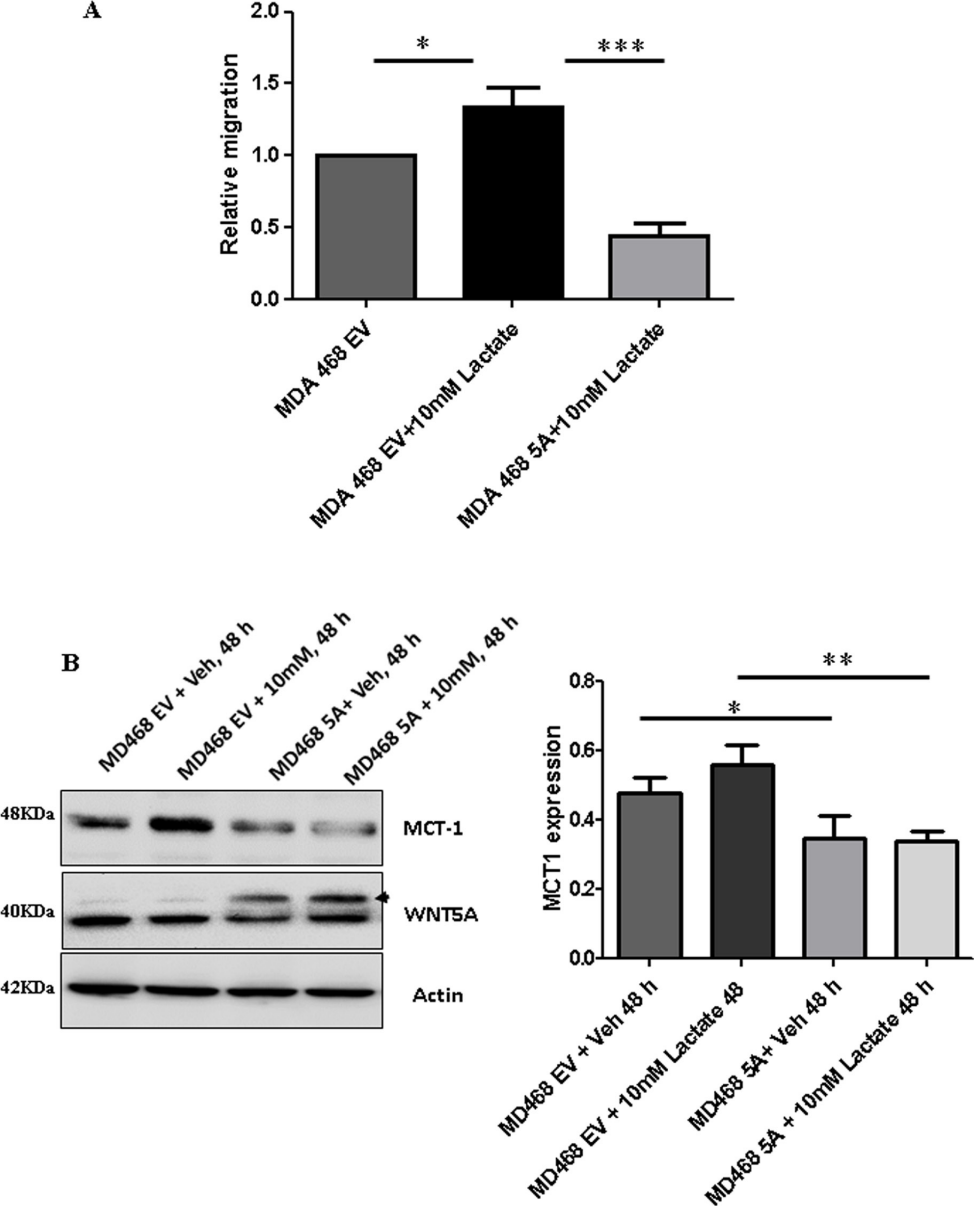
Lactate-induced migration of breast cancer cells is impaired in the presence of WNT5A signaling in MDA-MB-468 cells transfected with WNT5A (**A**) WNT5A-expressing breast cancer cells (MDA-MB-468-5A) and empty vector (MDA-MB-468-EV) cells were treated with 10 mM lactate or vehicle (water) for 48 h and then subjected to transwell migration analysis for the next 24 h. The experiments with WNT5A transfected breast cancer cells were performed for 72 h (end point) to allow sufficient time for secretion of WNT5A. All error bars represent the standard error of the mean (*n* = 4). *p < 0.05, ***p = 0.001. (**B**) Representative Western blot demonstrating the expression levels of MCT1 in MDA-MB-468 (MDA-MB-468-5A) and empty vector (MDA-MB-468-EV) cells treated with 10 mM lactate or vehicle (*left panel*). Actin served as a loading control. MCT-1 levels for the experiments were quantified by calculating integrated densitometric values and normalizing them to actin levels (*right panel*). The experiments were with WNT5A transfected breast cancer cells were performed for 72 h (end point) to allow sufficient time for secretion of WNT5A. All error bars represent the standard error of the mean (*n* = 3). *p < 0.05,**p = 0.01.

WNT5A signaling inhibits aerobic glycolysis in breast cancer cells via a β-catenin-PFKP axis resulting in reduced lactate production. However, in cancer tissue, lactate is not only generated from cancer cells but also from stromal cells in the cancer microenvironment. The presence of stromal-derived lactate in the tumor microenvironment can be imported into cancer cells through the specific lactate transporter, monocarboxylate transporter 1 (MCT1), and can consequently also regulate migration and invasion of such cancer cells. To study the effects of extracellular lactate on breast cancer cell migration in the absence and presence of WNT5A signaling, we treated breast cancer cells with sodium L-lactate (10 mM) under different conditions and evaluated their migratory responses. A significant increase in migration was found for lactate-treated MDA-MB-468-EV cells as compared to vehicle (water)-treated EV cells (Figure 7A). However, WNT5A-expressing MDA-MB-468 cells did not respond to extracellular lactate treatment, suggesting that WNT5A signaling abolishes the response of breast cancer cells towards extracellular lactate. In search of an underlying mechanism, we next investigated if WNT5A signaling affects the expression of the MCT1 lactate transporter in these cells. We found decreased expression of MCT1 in both MDA-MB-231-5A and MDA-MB-468-5A cells in comparison with their respective empty vector transfected control cells (Figure 7 and Supplementary Figure 5). As the endogenous expression of MCT1 was very low in MDA-MB-231 cells (Supplementary Figure 5) we performed the subsequent experiments in MDA-MB-468 cells. We therefore validated the decreased expression levels of MCT1 in MDA-MB-468-WNT5A cells compared to MDA-MB-468-EV cells and these results suggest that WNT5A signaling inhibits MCT1 expression (Figure 7B). Furthermore, even in the presence of lactate, breast cancer cells expressing WNT5A showed a statistically significant reduced level of MCT1 as compared to lactate treated MDA-MB-468-EV cells (*p* = 0.006) (Figure 7B).

**Figure 8:**
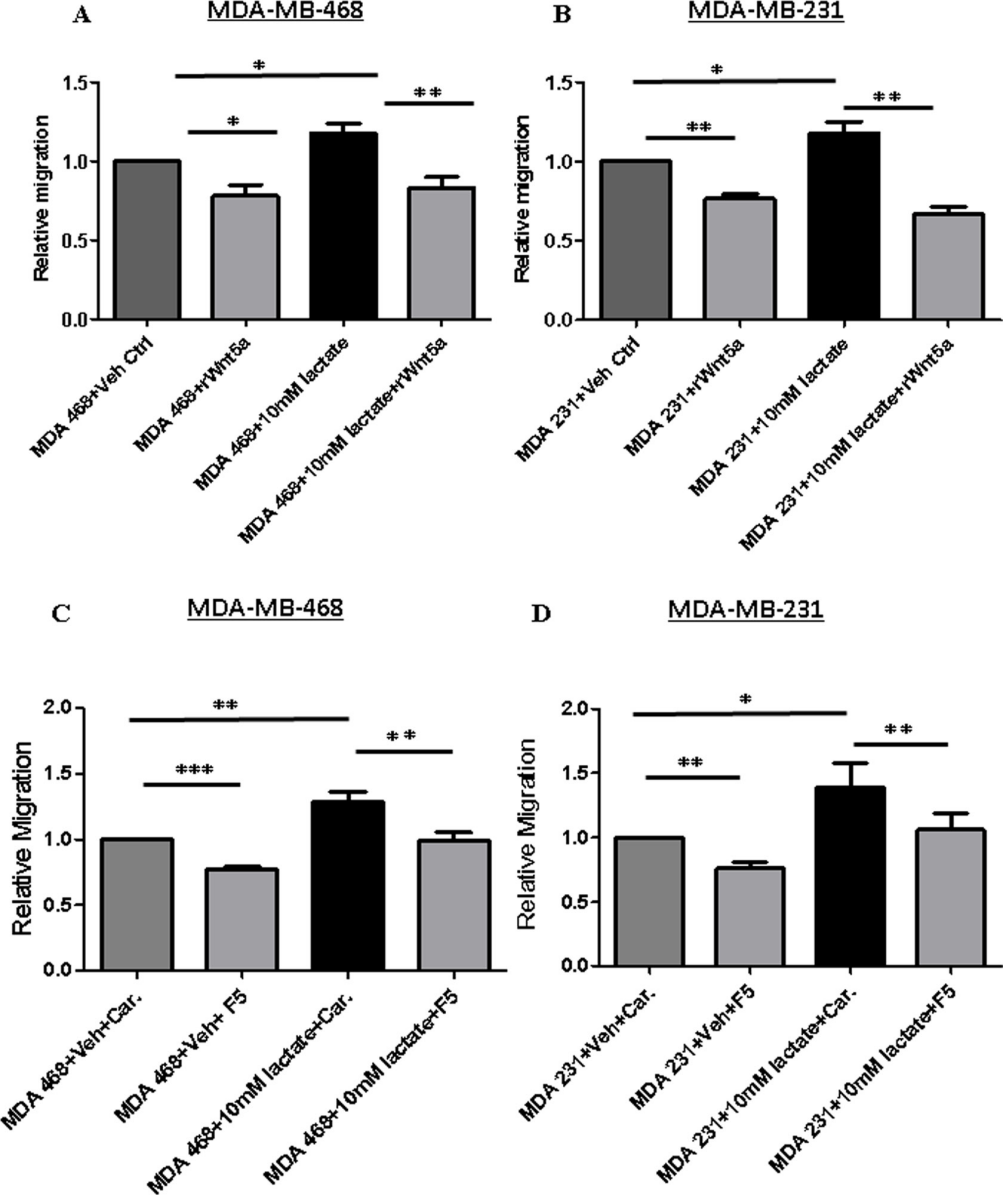
Lactate-induced migration of breast cancer cells impaired in MDA-MB-468 cells stimulated with rWNT5A or Foxy5 (**A**) MDA-MB-468 and (**B**) MDA-MB-231 breast cancer cells were treated with 10 mM lactate in the presence or absence of rWNT5A (0.4 μg/ml) for 24 h and then subjected to transwell migration analysis for the next 24 h. All error bars represent the standard error of the mean (*n* = 4). *p < 0.05, **p = 0.01. (**C**) MDA-MB-468 and (**D**) MDA-MB-231 breast cancer cells were treated with 10 mM lactate in the presence or absence of Foxy5 (100 μM) for 24 h and then subjected to transwell migration analysis for the next 24 h. These experiments were performed with parental breast cancer cell lines treated with lactate in the absence or presence of exogenously added recombinant WNT5A (0.4 μg/ml) or Foxy5 (100 μM). These experiments were performed for 48 h (endpoint) since we did not have to include time for secretion of WNT5A. All error bars represent the standard error of the mean (*n* = 4). *p < 0.05, **p = 0.01, ***p = 0.001.

These findings were further validated in non-transfected parental breast cancer cells (MDA-MB-468 and MDA-MB-231) treated with 10 mM L-lactate either in the absence or presence of rWNT5A or Foxy5 the WNT5A-mimic peptide (Figure 8). In accordance with our previous results (Figure 7), addition of exogenous lactate induced increased migration of both MDA-MB-468 and MDA-MB-231 parental breast cancer cells, and addition of either rWNT5A (Figure 8A and 8B) or Foxy5 (Figure 8C and 8D) caused a strong inhibition of migratory responses even in the presence of lactate in both cell lines.

## DISCUSSION

Cancer cells favor aerobic glycolysis as they can adjust themselves to fluctuating O_2_ tensions, while other cells that rely on oxidative phosphorylation to generate ATP might perish under such conditions [44]. In human breast cancer, the activity of glycolytic enzymes, such as hexokinase, pyruvate kinase and lactate dehydrogenase, is many fold higher compared to normal tissues [45–47]. In the current study, we investigated the role of WNT5A signaling in the regulation of aerobic glycolysis in breast cancer. Unlike melanomas in which WNT5A triggers tumor-promoting effects including a positive regulation of aerobic glycolysis, we expected an opposing effect in breast cancer cells where WNT5A is predominantly considered to exert tumor suppressor functions [20, 29, 40, 48, 49]. The breast cancer cell lines used in the present study (MDA-MB-468 and MDA-MB-231) both lack endogenous expression of WNT5A protein, making them ideal to study the effects of WNT5A signaling via either transfection with a WNT5A plasmid or direct stimulation with rWNT5A or Foxy5, a WNT5A-mimic peptide. Our results demonstrated that WNT5A signaling causes a reduced production of lactate in breast cancer cells in parallel with reduced cellular migration and down-regulation of PFKP. Our investigation also revealed that other isoforms of phosphofructokinase, i.e., *PFKL* and *PFKM*, do not play important roles in promoting breast cancer progression as only high PFKP gene expression is associated with poor survival in breast cancer patients. Using a siRNA knockdown approach, we demonstrated that PFKP plays a crucial role in regulating aerobic glycolysis in breast cancer cells. We demonstrated that PFKP-silenced breast cancer cells exhibited reduced lactate production as well as impaired breast cancer cell migration and invasion. In support of our findings, Park *et al*. demonstrated that the Tat-activating regulatory DNA-binding protein (TARDBP) regulates aerobic glycolysis by regulating PFKP through miR-520, suggesting that PFKP is indeed a key molecule in altering glucose metabolism [50]. Recent studies in which PFKP expression was indirectly increase or decrease either via the ZBTB7A transcriptional repressor or by sorafenib have suggested that PFKP might be a suitable drug target for anti-cancer therapies as inhibition of PFKP represses aerobic glycolysis [51, 52].

The question then arises via what pathway WNT5A decreases the expression of PFKP and thus glycolysis. Based on the findings that WNT5A can reduce β-catenin activity in colon cancer cells [23] and that β-catenin signaling induces aerobic glycolysis in breast cancer cells [8], we explored the possibility that WNT5A impairs aerobic glycolysis in breast cancer cells via impaired β-catenin signaling. For these experiments, we used an anti-active-β-catenin antibody and found that WNT5A signaling significantly reduced the amount of active-β-catenin protein in breast cancer cells. The data was further validated by demonstrating reduced LEF/TCF reporter activity in WNT5A-expressing breast cancer cells, proposing that WNT5A not only suppresses β-catenin protein expression but also inhibits its downstream transcriptional activity in breast cancer cells. The present observation that the inhibitor of β-catenin signaling, XAV939, impairs not only the amount of active β-catenin but also the expression of PFKP in a manner similar to that of WNT5A signaling supports the idea that WNT5A impairs aerobic glycolysis in breast cancer cells via reduced β-catenin signaling. Further support of this comes from the findings that XAV939 impairs both β-catenin signaling and PFKP expression more efficiently than WNT5A signaling. In support of our results and conclusions, Pate et al. recently demonstrated that XAV939 treatment of colon cancer cells reduces their glucose consumption and lactate production in parallel with a 30–40% increase in their oxidative phosphorylation rate [39].

**Figure 9:**
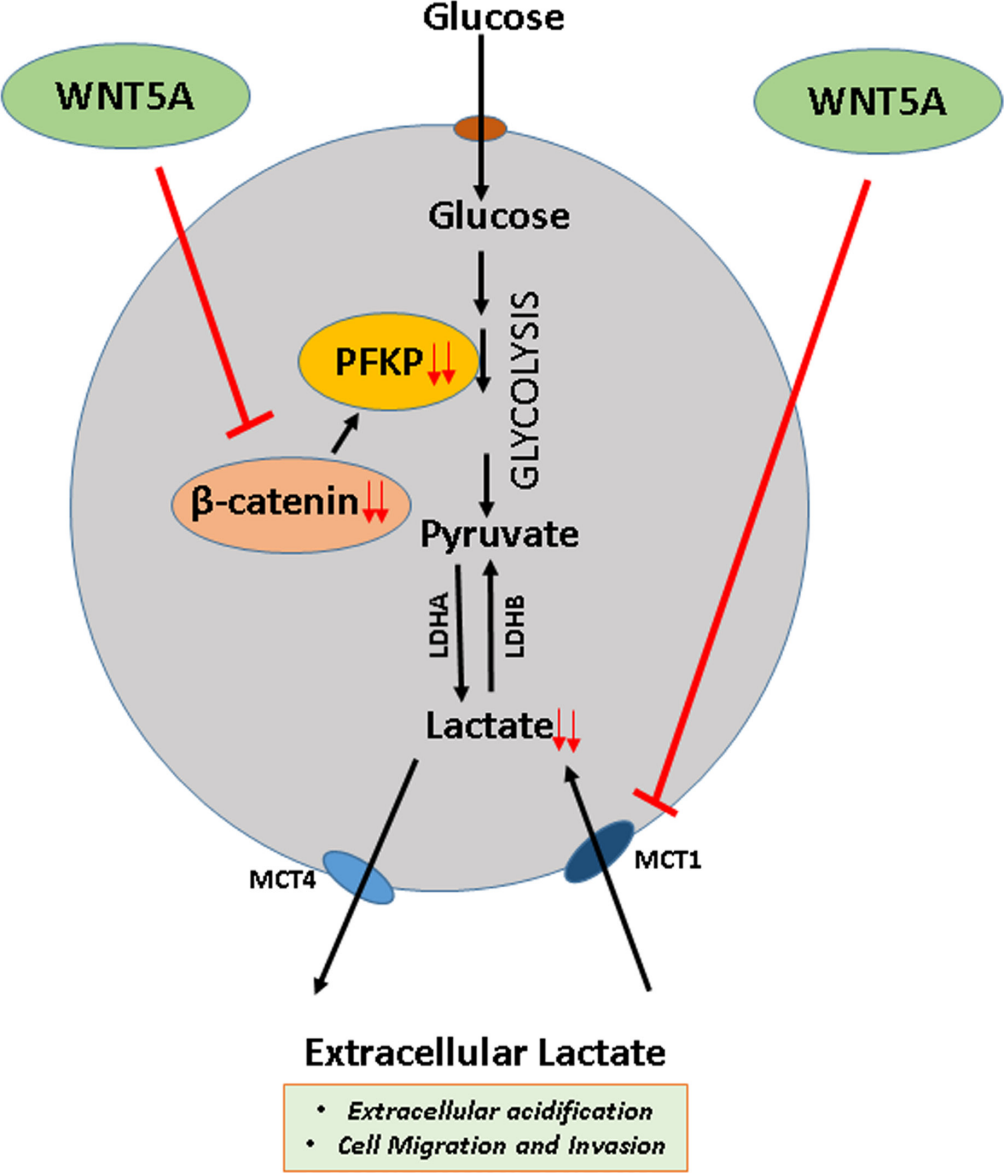
Schematic diagram of WNT5A signaling regulation of aerobic glycolysis in breast cancer cells The present study favors the idea that WNT5A signaling via a β-catenin-PFKP axis reduces lactate production and decreases the expression of MCT1 (a lactate uptake carrier) in breast cancer cells thereby adding to the ability of WNT5A to impair migration and invasion.

Cancer cells are not the only source of lactate in breast tumor tissue as stromal fibroblasts in the tumor microenvironment can also secrete lactate. By culturing mammary stromal cells (RMF-621) in high glucose conditions, Tobar *et al*. recently demonstrated a metabolic response characterized by induced lactate production in these cells that in turn fuels mammary epithelial growth with significant increase in cell migration and invasion [53]. In accordance with our present results, these authors also showed that lactate treatment alone significantly upregulates the transwell invasion of MDA-MB231 breast cancer cells [53]. Similarly, Bonuccelli *et al*. demonstrated that L-lactate stimulates MDA-MB-231 cells from metastatic lung lesions compared to control cells, suggesting that lactate drives the metastatic spread of breast cancer cells [54]. As we have shown previously that WNT5A signaling inhibits endogenous migration of breast cancer cells [40], we next investigated if active WNT5A signaling in breast cancer cells can counteract the effect of extracellular lactate. In the present study, we demonstrated that increased migration induced by extracellular lactate was impaired in WNT5A-transfected breast cancer cells, thereby suggesting that WNT5A might inhibit lactate uptake in breast cancer cells. Our finding that WNT5A signaling inhibits the expression of the key lactate importer, MCT1, suggests a mechanism whereby WNT5A counteracts the effect of extracellular lactate in breast cancer cells and thereby their ability to migrate and disseminate to form metastatic lesions. In support of our present finding that WNT5A impairs the expression of MCT1, inhibition of MCT1 has been shown to impair lactate transport as well as migration and invasion of breast cancer cells [55, 56]. On the basis of our results, we have provided a schematic diagram on how WNT5A signaling regulates aerobic glycolysis and thereby cell migration in breast cancer cells (Figure 9). Finally, our results demonstrating that both rWNT5A and Foxy5 continue to inhibit breast cancer cell migration, even in the presence of extracellular lactate, lends further support for the idea of therapeutically using Foxy5 to impair metastatic spread in the treatment of extremely glycolytic and aggressive breast cancers.

Overall, we showed that WNT5A signaling not only inhibits lactate production but also impairs lactate import, thereby inhibiting cell migration. Our study also highlighted the importance of WNT5A, β-catenin, PFKP and MCT1 in lactate regulation in breast cancer cells, thereby providing a therapeutic window where agonists (such as Foxy5) and antagonists of these molecules can be utilized for breast cancer therapy either alone or in combination.

## MATERIALS AND METHODS

### Cell lines

The MDA-MB-468 (Lot No. 58483213) and MDA-MB-231 (Lot No. 58629943) human mammary carcinoma cell lines were procured directly from the American Type Culture Collection. All cell lines were grown in DMEM (HyClone, Utah, USA) supplemented with 10% FBS, 5 U/ml penicillin, 0.5 U/ml streptomycin, and 2 mM glutamine. The cell lines were frequently screened for mycoplasma contamination using an EZ-PCR kit (HaEmek, Israel).

### Plasmid and siRNA transfections

The pcDNA3.1 (±)-WNT-5A plasmid or the pcDNA3.1 empty vector were used to transfect the MDA-MB-468 and MDA-MB-231 breast cancer cells lines as described by Prasad *et al*. [40]. The stable breast cancer cells expressing WNT-5A (i.e., MDA-MB-468-5A and MDA-MB-231-5A) were maintained in DMEM supplemented with 10% FBS and 700 μg/mL geneticin (G418). Continued WNT-5A expression was verified by Western blot analysis performed on cell lysates and on conditioned cell culture media. Prior to the lactate assay, proliferation assay, migration/or invasion assay, LEF/TCF reporter assay, siRNA transfections and Western blot analysis, the cells were washed with PBS and grown for 48 h in G418-free DMEM supplemented with 1% FBS to condition the media with WNT-5A.

For PFKP transient knockdown, sequence-specific siRNAs were used. The smart pool of PFKP siRNA (h) (Cat. no.sc-106401) was procured from Santa Cruz Biotechnology Inc. (Texas, USA). The negative control (NC) scrambled sequence #1 siRNA was obtained from Applied Biosystems (CA, USA). Briefly, breast cancer cells were transiently transfected with a transfection complex of 25 nM siRNA (final concentration) and Lipofectamine 2000 transfection reagent (Invitrogen, CA, USA) suspended in serum-free DMEM. After 6 h, the transfection complex was removed, and the cells were replenished with fresh DMEM containing 10% FBS. The cells were then allowed to grow for the next 48 h prior to use for Western blot analysis, lactate assay, migration assay or invasion assay.

### Western blotting

WNT5A-expressing breast cancer cells or parental breast cancer cells treated with PFKP siRNA, rWNT5A, XAV939, U0126 or not were washed with ice-cold PBS and lysed in ice-cold phosphorylation lysis buffer (PLB). The protein estimation, SDS-PAGE and visualization procedures were performed as described in Prasad *et al.* [40]. The following primary antibodies were used: anti-WNT5A from R&D systems (MN, USA); anti-Hexokinase-II, anti-Puruvate Kinase; anti-PKFP, anti-non-phospho (Active) β-catenin, and anti-pERK1/2 antibodies from Cell Signaling Technology (MA, USA); anti-MCT1 antibody from Santa Cruz Biotechnology Inc. (TX, USA); and anti-β-actin antibody from Sigma-Aldrich (MO, USA). The secondary antibodies used were goat anti-mouse, goat anti-rabbit and rabbit anti-goat, which were procured from Dako (Glostrup, Denmark). Separated protein bands were visualized using Chemiluminescence HRP substrate (Millipore), and the membranes were imaged and analyzed using the Chemi Doc™ imaging system from Bio-Rad.

### Kaplan-meier survival analysis

The Kaplan-Meier-Plotter online software was used to perform meta-analysis-based biomarker prediction as described by Gyorffy *et al*. [31]. For our analysis, we plotted and calculated overall survival (OS) for breast cancer patients (*n* = 1117) with respect to expression of all the three isoforms of phosphofructokinase (*PFKP*, Platelet; *PFKL*, Liver; and *PFKM*, Muscle) and *WNT5A*.

### Lactate assay

Secreted lactate in the cell culture supernatant (from WNT5A-expressing breast cancer cells after siRNA PFKP or XAV939 treatment) was measured using the lactate estimation kit (BioVision) according to the manufacturer's instructions. All the experiments for lactate estimation were performed in low serum conditions.

### Cell proliferation assay

Briefly, WNT5A-expressing breast cancer cells (MDA-MB-468-5A and MDA-MB-231-5A) were seeded (5.0 × 10^4^ cells/well) in 12 well plates and were allowed to grow for 72 h. A 3-(4,5-dimethylthiazol-2-yl)-2,5-diphenyl tetrazolium (MTT) assay was used to assess cell proliferation. MTT solution (25 μl; 5 mg/ml) was added to each well 6 h prior to the end point. After 72 h, the medium was discarded and DMSO was added to the cells. Absorbance at 570 nm was measured using a microplate reader (FLUOstar Omega).

### Transwell migration and invasion assay

PFKP siRNA-transfected or L-lactate-treated breast cancer cells were washed and detached with Versene (Gibco^®^, NY, USA) and re-suspended as single-cell suspensions in low-serum DMEM (supplemented with 1% FBS). The cell invasion assays were performed in cell culture inserts (PET membrane with 8.0 μm pore size; Ref# 353097; Corning Incorporated, NC, USA). The cells were counted using an automated cell counter (Countess™, Invitrogen). A total of 25,000 cells in low-serum DMEM was added to the upper chamber [for rWNT5A treatment, 0.4 μg/ml rWNT5A was added to the medium], and the lower chamber was filled with 0.7 ml of DMEM supplemented with 10% FBS. The cells were allowed to invade for 24 h at 37°C in a humidified incubator with 5% CO_2_. After incubation, the cells in the insert were fixed with 4% paraformaldehyde for 10 min at RT. The non-migratory cells on the upper side of the insert were removed with a cotton-tipped applicator and were further processed as published by Linnskog *et al*. [57]. Transwell invasion assays for PFKP siRNA-treated MDA-MB-231 cells were performed in a similar manner using BD Matrigel™ invasion chambers (MA, USA) in which 50,000 cells were added to the upper chamber. Statistical analyses were performed by taking those cells into account that have migrated or invaded to the other side of the membrane (outer side). Furthermore, it was checked that the incubation time (24 h) used in these experiments did not allow cells to detach from the membrane into the lower chamber.

### LEF/TCF reporter assay

The LEF/TCF reporter assay was used to determine β-catenin-dependent WNT signaling activity. A construct containing a promoter with TCF-binding motifs upstream of a *Firefly* luciferase gene was transfected into the MDA-MB-468-5A and MDA-MB-468-EV cells. β-catenin binding to the TCF motif resulted in the transcription and translation of luciferase and in the emission of a bioluminescence signal. To normalize for transfection efficiency, a CMV promoter-driven *Renilla* luciferase reporter was co-transfected. The luciferase substrates were purchased from Promega (Dual-Luciferase^®^ Reporter (DLR) Assay, E1910) and automatically added by a dual-injector system according to manufacturer's protocol. Luciferase activity of cell extracts was measured using a Mini Lumat LB 9506 (Berthold Technologies).

### Immunofluorescence

Breast cancer cells (MDA-MB-468-EV & MDA-MB-5A) were grown on 13-mm glass cover-slips for 24 h at 37°C in a humidified atmosphere of 5% CO_2_. After incubation, cells were washed in PBS, fixed with 4% paraformaldehyde for 20 min, and permeabilized with 0.1% TritonX-100 for 3 min, all at room temperature. Next, cells were incubated with Phalloidin-TRITC (1:400; Sigma Aldrich) for 45 min at room temperature. Finally, the cells were rinsed with PBS and counter-stained with DAPI (300 nM in PBS) for 2 min. For Confocal imaging (Carl Zeiss LSM 700), the fixed and stained cells were mounted with the Dako Fluorescent Mounting Medium and viewed with a 40× objective.

### Statistical analysis

All the data presented herein are expressed as the mean ± standard error. Each of the experiments was repeated at least three times. Statistical analysis was throughout performed using two-tailed Student's *t*-test and *p* values < 0.05 were considered significant. For the lactate production experiments we used unpaired *t*-test and for the migration and invasion assays we used paired *t*-test. All of the statistical tests and graphs were generated using GraphPad Prism 5.0 software (CA, USA).

## SUPPLEMENTARY MATERIALS FIGURES



## Abbreviations

PFKP: Phosphofructokinase platelet-type
rWNT5A: recombinant WNT5A
MCT1: monocarboxylate transporter 1
ERK1/2: Extracellular Signal-Regulated Kinases 1 and 2
