# Supplementary material for: Reduced production and uptake of lactate are essential for the ability of WNT5A signaling to inhibit breast cancer cell migration and invasion
Source: Oncotarget. 2017 Apr 20;8(42):71471–88. doi: 10.18632/oncotarget.17277 (PMC5641063; doi:10.18632/oncotarget.17277)
Supplement: Supplementary file 1 [file oncotarget-08-71471-s001.pdf]

Reduced production and uptake of lactate are essential for the ability of WNT5A signaling to inhibit breast cancer cell migration and invasion

Supplementary Materials

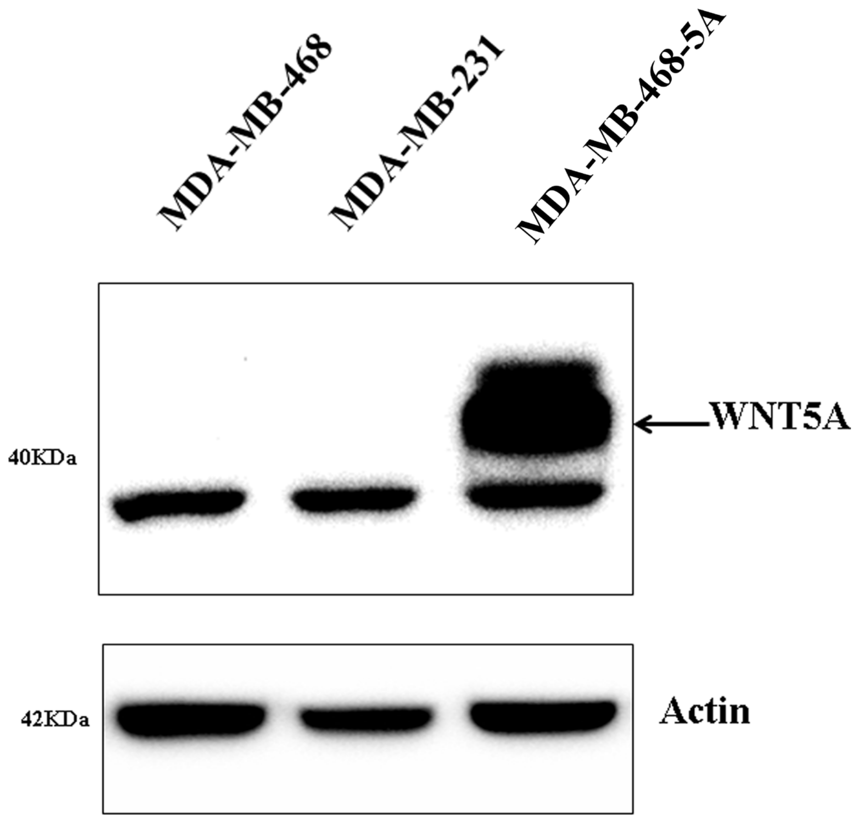

Supplementary Figure 1: WNT5A expression in triple negative breast cancer (TNBC) cells. Representative Western Blot, Lane 3 showing the presence of WNT5A protein in whole lysates from MDA-MB-468 and MDA-MB-231 breast cancer cells ($n = 3$). MDA-MB-468 cells stably transfected with the WNT5A plasmid (MDA-MB-468-5A) was used as a positive control for the experiment. Actin was used as a loading control.

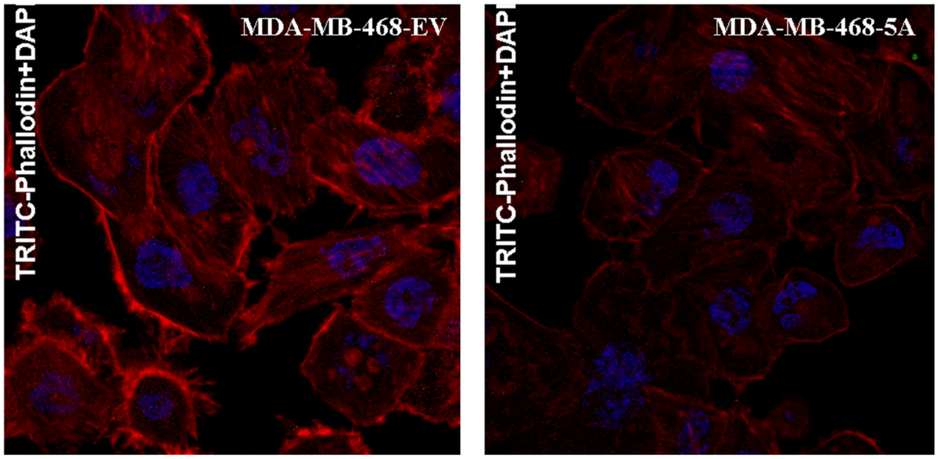

Supplementary Figure 2: Morphological analysis of MDA-MB-468-5A cells via Phalloidin-TRITC staining. WNT5A expressing MDA-MB-468 cells (MDA-MB-468-5A, *Right Panel*), and control cells (MDA-MB-468-EV, *Left Panel*) were grown on 13-mm glass coverslips for 48 h, followed by Immunofluorescence procedure as described in Materials and Methods. Significant migration relevant membrane protrusions were observed in MDA-MB-468-EV cells, compared to MDA-MB-468-5A cells.

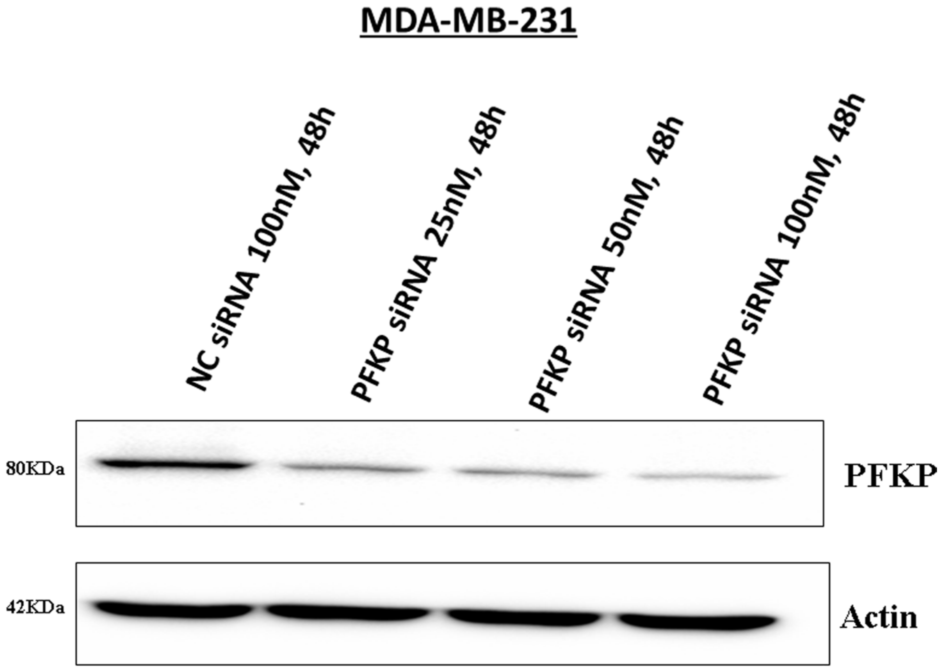

Supplementary Figure 3: Optimization of PFKP siRNA in MDA-MB-231 breast cancer cells. Representative Western blot of MDA-MB-231 cells transfected with PFKP siRNA at varying concentrations (25, 50 and 100 nM) for 48 h.

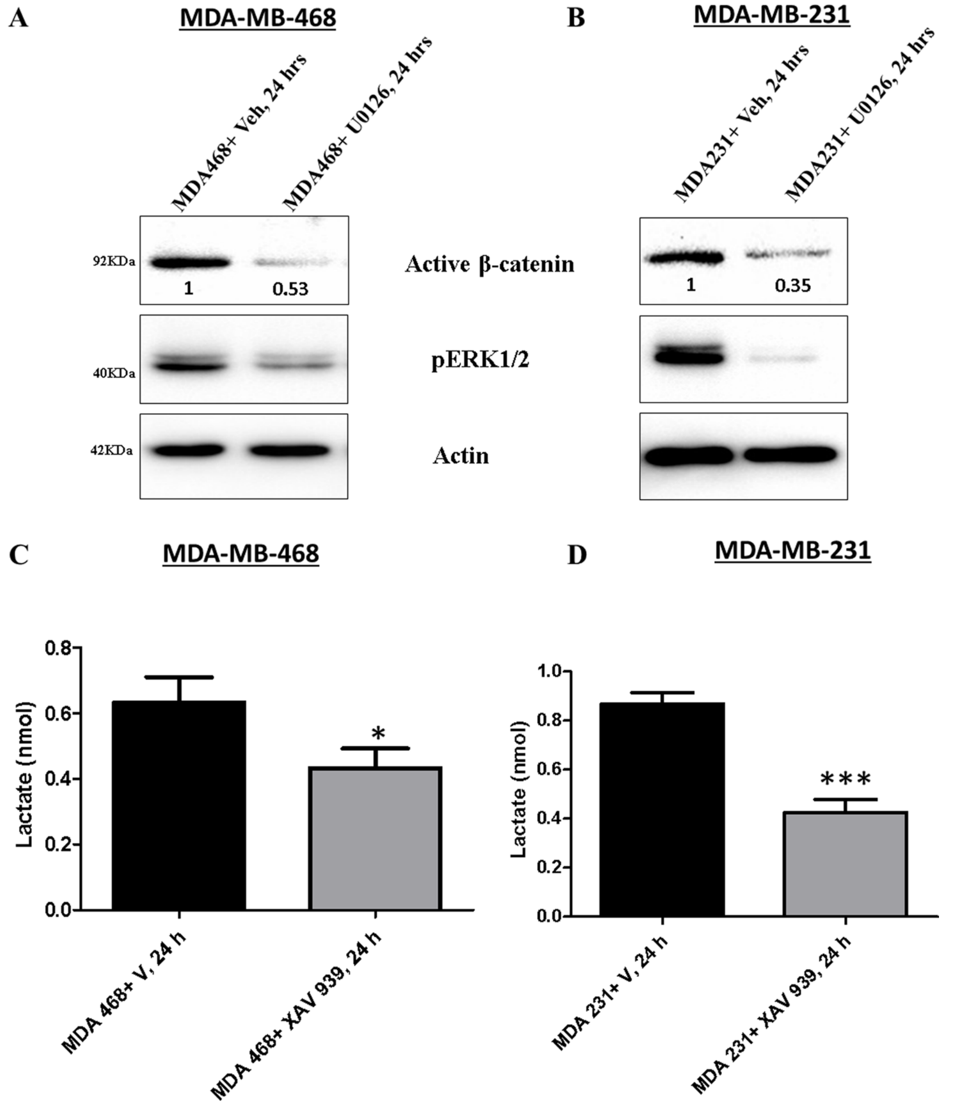

Supplementary Figure 4: U0126 and XAV939 (an inhibitor of β-catenin) inhibit lactate production. (**A**) MDA-MB-468 and (**B**) MDA-MB-231 breast cancer cells were treated with U0126 (10 μM) or left untreated for 24 h, and Western blotting was used to determine active β-catenin expression. The expression of pERK1/2 was also analyzed as an experimental control. Extracellular lactate levels in (**C**) MDA-MB-468 and (**D**) MDA-MB-231 breast cancer cells treated with XAV939 (10 μM) for 24 h were evaluated. All error bars represent the standard error of the mean ($n = 4$). $*p < 0.05$, $**p = 0.01$.

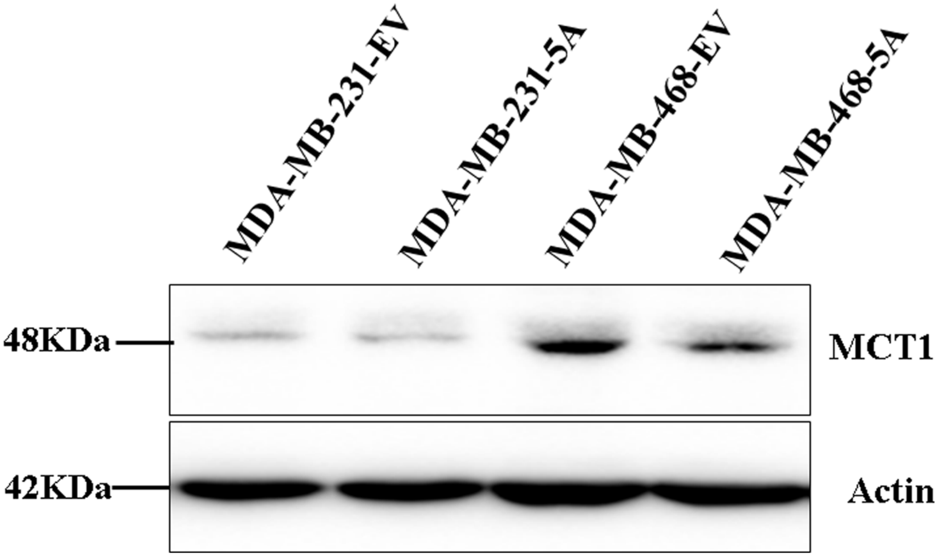

Supplementary Figure 5: MCT1 expression in WNT5A transfected breast cancer cell lines. Representative Western blot demonstrating the expression levels of MCT1 in MDA-MB-231-5A and MDA-MB-468-5A, as compared to their respective empty vector (EV) cells. Actin served as a loading control. The experiments were performed with WNT5A transfected breast cancer cells for 72 h (end point) to allow sufficient time for secretion of WNT5A.
